# Case Report: Discordant pancreatic inflammatory pseudotumor in a breast cancer survivor – a diagnostic dilemma

**DOI:** 10.3389/fonc.2026.1767121

**Published:** 2026-03-23

**Authors:** Zhe Cao, Xiaohong Lyu, Xiaoyue Lu, Taiping Zhang

**Affiliations:** Department of General Surgery, Peking Union Medical College Hospital, Chinese Academy of Medical Sciences & Peking Union Medical College, Beijing, China

**Keywords:** abscopal effect, breast cancer, immunotherapy, pancreatic lesion, radiation therapy

## Abstract

**Background:**

Differentiating suspicious lesions presents a clinical challenge due to overlapping characteristics among benign, malignant, primary, and secondary pathologies, often leading to diagnostic errors and inappropriate treatment.

**Case presentation:**

We report on a 54-year-old patient with triple-negative breast cancer (TNBC) who, during postoperative surveillance, was found to have pancreatic lesions. Although initial imaging and biopsy suggested adenocarcinoma, surgical excision and subsequent histopathological examination revealed chronic pancreatitis with atypical hyperplasia.

**Conclusion:**

This case highlights the limitations of preoperative diagnostics and the complexity of distinguishing treatment-related injury from malignant recurrence, particularly in patients with prior exposure to radiotherapy and immunotherapy. To mitigate overtreatment, we advocate for a diagnostic paradigm that integrates systematic screening, advanced molecular profiling (e.g., next-generation sequencing), and a rigorous, multidisciplinary evaluation for all cancer survivors.

## 1 Introduction

Pancreatic cancer, a leading cause of mortality, is aggressive and frequently diagnosed at a late stage. Patients with a history of breast cancer have an elevated risk of pancreatic metastases, which account for about 2% of all pancreatic malignancies and are more common in aggressive subtypes like triple-negative breast cancer (TNBC) (1). The diagnostic challenge in these patients is compounded by overlapping imaging and histopathological features among primary tumors, metastatic disease, and benign conditions. Standard tools like imaging and fine-needle biopsy can be ambiguous due to atypical inflammatory changes, risking unnecessary, aggressive interventions that increase morbidity. A careful, systematic, and multidisciplinary evaluation is therefore essential to optimize patient outcomes, particularly for those with complex treatment histories. This report highlights these challenges and the critical value of such a multidisciplinary approach.

## 2 Case presentation

### 2.1 History and examination

**Figure 1:**
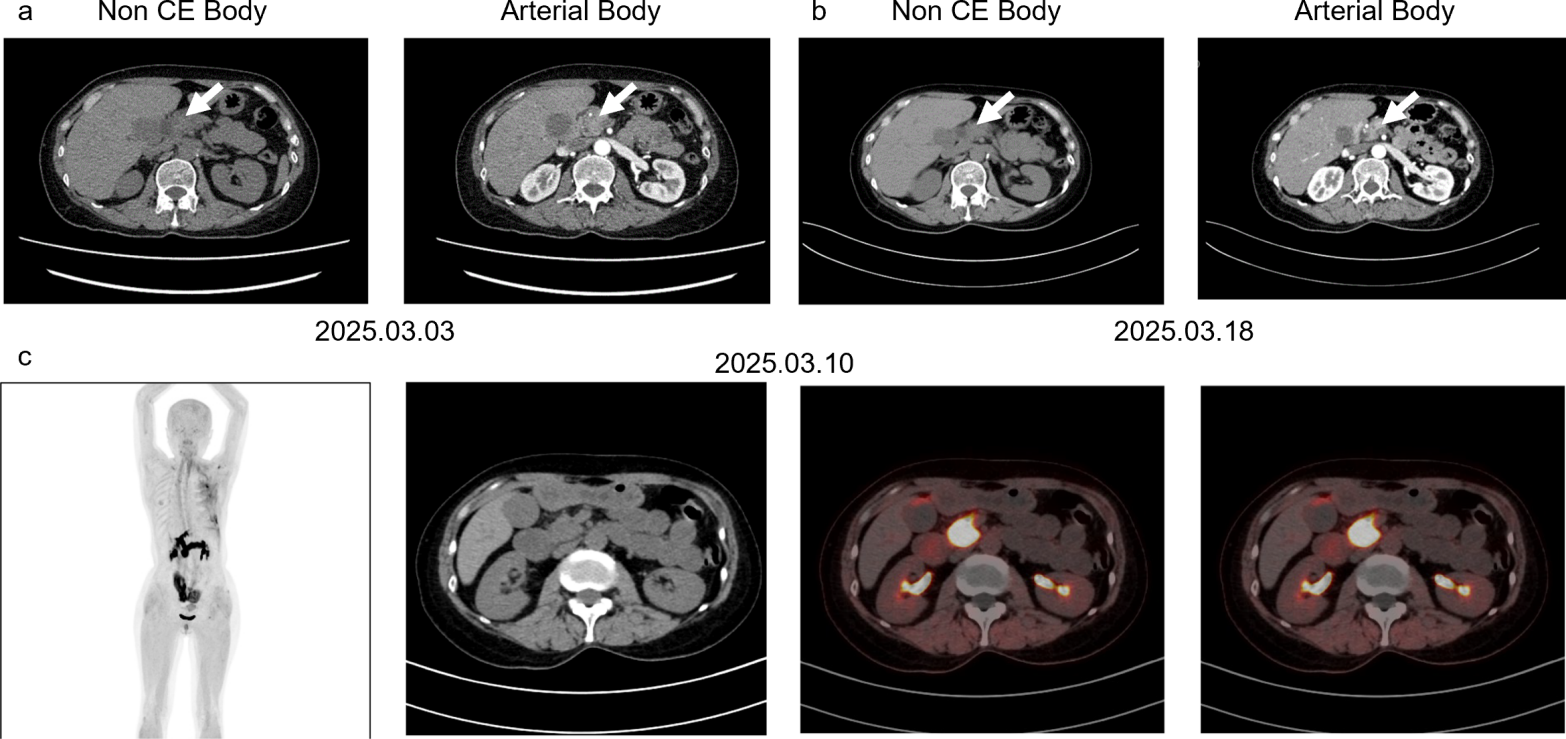
Radiological results for this patient. **(a, b)** are two CT results in March 2025. On March 1, 2025, an ill-defined, swollen mass was identified in the pancreatic head without appreciable delayed-phase enhancement. On March 18, 2025, the lesion was more clearly delineated, with reduced surrounding swelling and progressive delayed-phase enhancement, suggesting a substantial fibrous component and suspicious malignancy. **(c)** is the FAPI PET-CT result.

**Figure 2:**
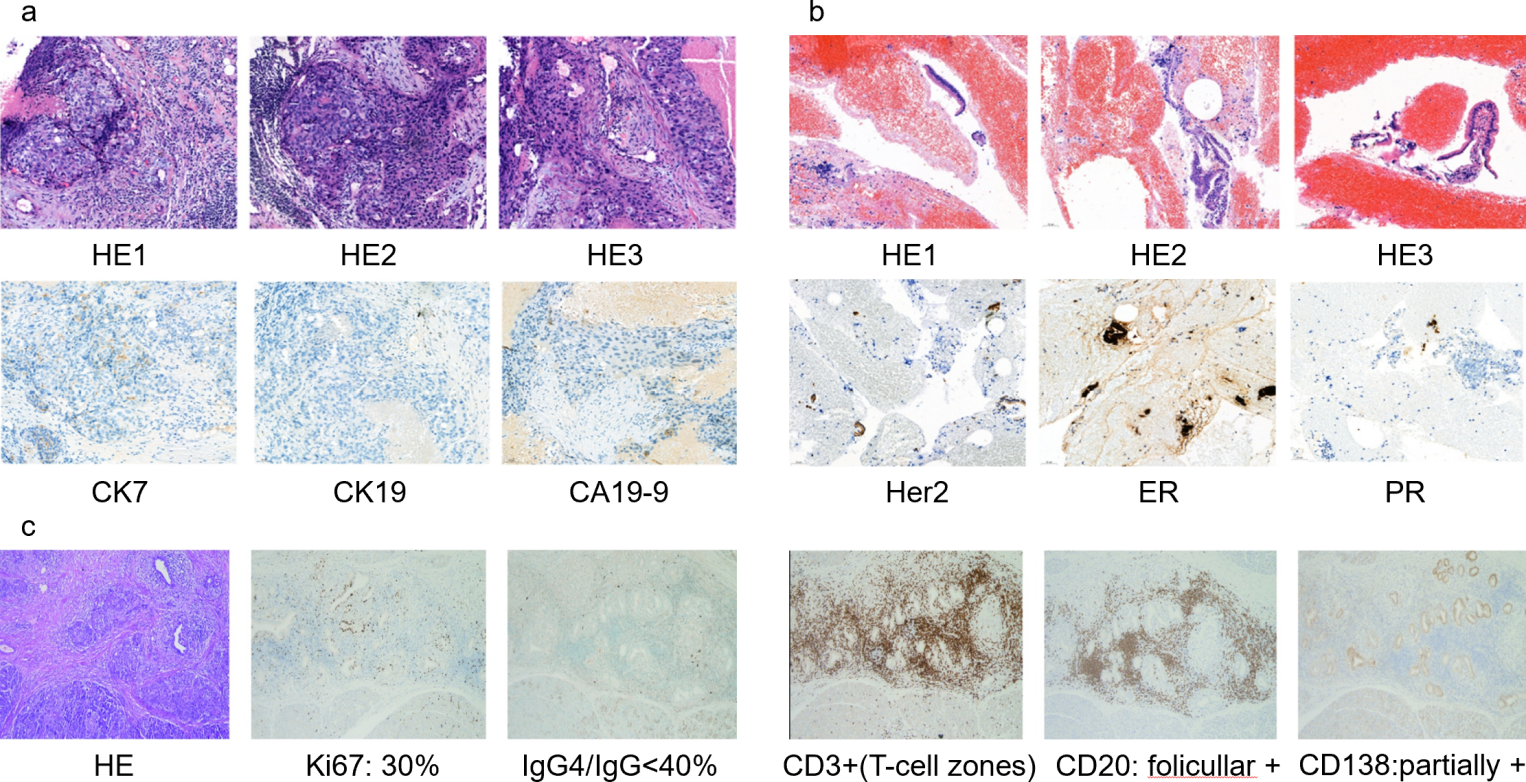
Pathological results for this patient. **(a–c)** are respectively for the fine needle biopsy specimen in 2023.12, the EUS-FNA specimen in 2025.02, and the pancreatoduodenectomy specimen.

During a routine surveillance CT in December 2024, a 54-year-old female was found to have pancreatic lesions (**Figure 1a**). Initial endoscopic ultrasound with fine-needle aspiration (EUS-FNA) on December 25, 2024, revealed atypical cellular changes. A subsequent PET/CT on January 24, 2025, showed a hypermetabolic focus (SUVmax 8.2) in the pancreatic head/neck, raising suspicion of malignancy. Secondary EUS-FNA on February 17, 2025, diagnosed adenocarcinoma of indeterminate origin, which was confirmed by an expert histopathology review on February 25, 2025. Immunohistochemical (IHC) staining was negative for breast-specific markers (GATA3, mammaglobin, GCDFP-15) but positive for CK7, CK20, and Villin (**Figure 2b**). Subsequent MRI on March 1, 2025, identified a 1·9 cm x 2·5 cm irregular pancreatic mass. A FAPI PET/CT on March 10, 2025, and a pancreatic CT on March 18, 2025, also showed intense tracer uptake (**Figures 1b, c**).

In December 2023, the patient was diagnosed with a left breast mass on routine ultrasonography. Core-needle biopsy confirmed invasive, poorly differentiated carcinoma with triple-negative receptor status (ER 0%, PR 0%, HER2 negative) and a high proliferative index (Ki-67 80%) (**Figure 2a**, **Supplementary Figure 1b**). She completed six cycles of neoadjuvant chemotherapy (December 2023–April 2024; AP + PD-L1 regimen every 21 days) followed by breast-conserving radical mastectomy in April 2024. The postoperative pathology report showed a pathological complete response (pCR; ypT0/Tis, ypN0, 8th edition AJCC), with no residual invasive carcinoma in the breast or axillary lymph nodes (0/15). Comprehensive genomic profiling (NGS) of the surgical specimen in January 2024 identified no clinically significant BRCA1/2 mutations. Adjuvant therapy included locoregional radiotherapy and maintenance immunotherapy. Radiotherapy (May–June 2024) was delivered to the tumor bed and regional lymphatics at a total dose of 50 Gy in 25 fractions. Immunotherapy with the PD-L1 inhibitor adebrelimab (20 mg/kg IV every 3 weeks) began concurrently with neoadjuvant chemotherapy and continued for 11 cycles to December 2024.

### 2.2 Diagnosis and management

Preoperative imaging and biopsy suggested a malignant pancreatic head lesion, but its origin was uncertain. The negative results for IgG subclasses had largely removed the possibility of autoimmune pancreatitis (**Supplementary Table 5**). The multidisciplinary team considered primary pancreatic carcinoma versus metastatic breast disease. Surgery was considered curative for a primary tumor but palliative for metastasis, with multimodality therapy as an alternative. Following a multidisciplinary consultation and the patient’s request, surgical resection was recommended.

The patient underwent an open pancreatoduodenectomy. Postoperative course was uneventful, with no complications such as pancreatic fistula, hemorrhage, or surgical site infection. Enteral feeding was advanced on postoperative day (POD) 7, and the patient was discharged on POD 10, with no 30-day readmissions. Final histopathology revealed chronic fibroinflammatory pancreatitis, including acinar atrophy, ductal epithelial hyperplasia and dysplasia, and microabscesses with lymphocytic and neutrophilic infiltrates. All surgical margins were free of neoplasia. The gallbladder showed chronic cholecystitis, and all 44 examined lymph nodes were negative for metastasis (pN0).

Immunohistochemical and ancillary studies confirmed an inflammatory, non-neoplastic process. Ki-67 staining showed 30% nuclear positivity in the residual ductal epithelium, while lymphoid markers revealed diffuse CD3 positivity (T-cell zones) and follicular CD20 staining (B-cells). Plasma cell markers were partially positive for CD138, with 5 IgG4+ plasma cells per high-power field, resulting in an IgG4/IgG ratio of less than 40%. P53 staining was wild-type with scattered positivity. Finally, CK7 strongly stained the ductal epithelial structures, confirming their epithelial lineage but not supporting a diagnosis of metastatic breast carcinoma (**Figure 2c**). These findings reclassified the lesion as chronic pancreatitis with atypical reactive epithelial changes, necessitating a reassessment of the preoperative diagnosis.

## 3 Discussion

**Figure 3:**
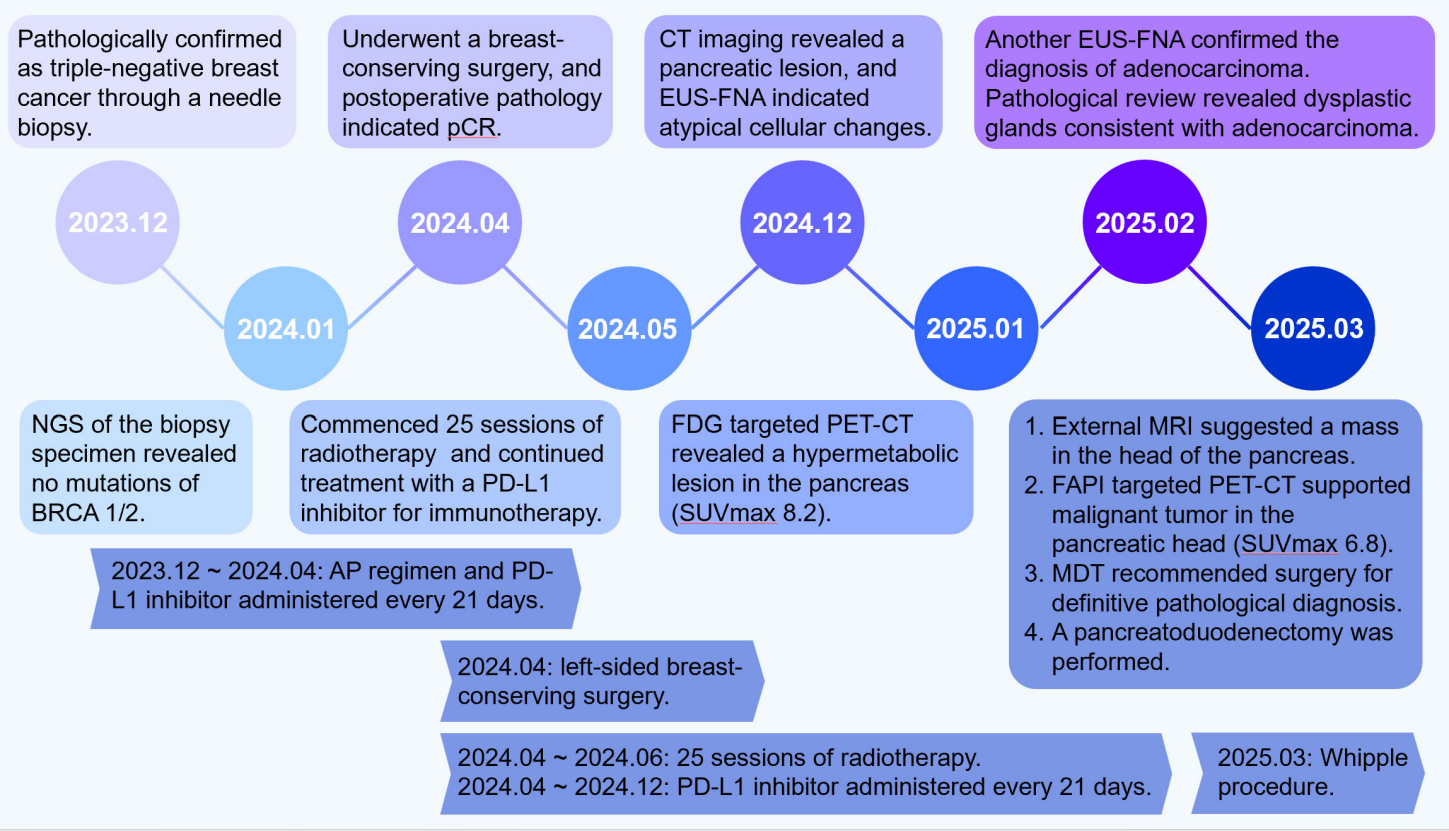
A concise timeline of the patient’s clinical course, diagnostic events, and therapeutic interventions is presented in a schematic format below.

Our case highlights a critical diagnostic challenge: a TNBC survivor whose pancreatic lesions mimicked malignancy across multimodal imaging (FDG-PET/CT, FAPI-PET/CT, and MRI) and initial biopsy but were ultimately confirmed as chronic pancreatitis with atypical hyperplasia (**Figure 3**). This underscores the potential for inflammatory and post-therapeutic remodeling to perfectly simulate adenocarcinoma, leading to unnecessary surgical intervention.

While CT, HRCT, and MRI offer good specificity and sensitivity, their accuracy is compromised by small lesions or inflammatory backgrounds (2). ^18^F-FDG PET/CT is a standard in oncology, but its specificity is frequently compromised by inflammatory false positives. More notably, this patient’s FAPI-PET/CT (SUVmax 6.8) also favored malignancy. While Fibroblast Activation Protein (FAP) is highly expressed by cancer-associated fibroblasts (CAFs), it is also up-regulated in activated fibroblasts during chronic inflammation and tissue remodeling. This case serves as a cautionary reminder that FAPI-PET/CT, despite its high sensitivity for desmoplastic tumors, cannot reliably distinguish between tumor-induced desmoplasia and treatment-induced fibrosis.

The diagnostic uncertainty was compounded by the limitations of EUS-FNA. Sampling bias and reactive atypia often lead to false-positive cytologic interpretations. Furthermore, the absence of breast markers (GATA3) and the presence of a focal gastrointestinal phenotype (CK7^+^/CK20^+^/Villin^+^) suggested a new primary pancreatic cancer rather than a reactive process. This highlights the ‘double-edged sword’ of lineage-restricted IHC in the setting of inflammatory atypia, where reactive cells can lose or aberrantly express markers. While NGS could have provided clarity by identifying the absence of canonical KRAS or TP53 mutations, limited tissue volume remains a significant hurdle in EUS-guided diagnostics.

The most striking aspect of this case is the development of pancreatic pathology following chest wall radiotherapy and anti-PD-L1 therapy. While radiotherapy is traditionally viewed as a local modality, it can elicit systemic effects. We propose that the patient experienced a systemic inflammatory response, potentially through a mechanism analogous to the abscopal effect. The emerging phenomenon refers to tumor regression occurring not only at irradiated sites but also at distant, non-irradiated lesions (3), indicating that localized radiotherapy can elicit systemic antitumor immunity.

Localized radiation can release damage-associated molecular patterns (DAMPs) and activate the cGAS-STING pathway, promoting systemic immune activation (4, 5). In some instances, the release of pro-inflammatory cytokines like PAI-1 from irradiated stroma can modulate the distant microenvironment, recruiting myeloid cells to non-irradiated sites (6). When combined with immune checkpoint inhibitors (ICIs), this may exacerbate immune-related adverse events (irAEs) in non-target organs (7). Although radiation-induced pancreatitis is rare, the synergistic interaction between RT and ICIs likely created a ‘pro-inflammatory storm’ that manifested as chronic pancreatitis and atypical hyperplasia in the pancreas.

First proposed by Mole in 1953, the abscopal effect has been reported in only a limited number of metastatic solid tumors, including melanoma and renal cell carcinoma (8). Abscopal events typically manifest 2 to 6 months after RT, coinciding with the window in which our patient developed pancreatic changes (9). The classic abscopal case reported by Postow involved a patient on ipilimumab receiving RT to a paraspinal mass. Not only did distant hilar nodes regress, but the patient developed systemic vitiligo, which is a classic ‘off-target’ inflammatory complication signifying a systemic breakdown of immune tolerance (10). Nevertheless, the existence and clinical relevance of the abscopal effect remain debated. For example, a randomized phase II trial in metastatic head and neck squamous cell carcinoma demonstrated that the addition of stereotactic body radiotherapy (SBRT) to nivolumab did not significantly improve objective response rates (11).

## 4 Conclusion

To our knowledge, this is the first report of combined chemo-radiotherapy and immunotherapy associated with pancreatic inflammation mimicking malignancy. As cancer survivorship and immunotherapy use increase, clinicians must be alert to treatment-related inflammatory lesions mimicking malignancy. This case highlights the diagnostic challenges of complex patient histories and prior therapies, and the risk of equating radiological suspicion with histological certainty. The diagnostic challenge was further compounded by the rarity of abscopal effects from combined radiotherapy and immunotherapy in a non-metastatic site. We propose a three-step diagnostic framework for patients with prior radiotherapy or immunotherapy: (1) critically re-evaluate imaging in the context of treatment history; (2) use advanced tools like liquid biopsy or transcriptomic analysis to distinguish clonal neoplasia from reactive lesions; and (3) incorporate a multidisciplinary consultation to assess the necessity of surgical intervention. Integrating treatment history, molecular profiling, and multidisciplinary expertise may protect patients from the dual burden of malignancy and therapy-induced injury.

## Data Availability

The original contributions presented in the study are included in the article/**Supplementary Material**. Further inquiries can be directed to the corresponding author.

## Glossary

AJCC: American Joint Committee on Cancer
CAF: cancer-associated fibroblast
cGAS–STING: cyclic GMP–AMP synthase–stimulator of interferon genes
CK: cytokeratin
CT: computed tomography
DAMPs: damage-associated molecular patterns
EUS-FNA: endoscopic ultrasound–guided fine-needle aspiration
FAP: fibroblast activation protein
FAPI: fibroblast activation protein inhibitor
FDG: fluorodeoxyglucose
HRCT: high-resolution computed tomography
ICI: immune checkpoint inhibitor
IgG4: immunoglobulin G4
IHC: immunohistochemistry
irAE: immune-related adverse event
MRI: magnetic resonance imaging
NGS: next-generation sequencing
PAP: plasminogen activator protein
PAI-1: plasminogen activator inhibitor-1
PD-1: programmed cell death protein 1
PD-L1: programmed death-ligand 1
pCR: pathological complete response
PET: positron emission tomography
SUVmax: maximum standardized uptake value
TNBC: triple-negative breast cancer
