# Supplementary material for: Case Report: Discordant pancreatic inflammatory pseudotumor in a breast cancer survivor – a diagnostic dilemma
Source: Front Oncol. 2026 Mar 23;16:1767121. doi: 10.3389/fonc.2026.1767121 (PMC13050720; doi:10.3389/fonc.2026.1767121)

**Supplementary Material**

Supplementary tables 1 to 6 are the preoperative serum test results of this patient. Hematologic and biochemical testing revealed no absolute contraindications to surgery. Inflammatory markers were within reference ranges, and tumor markers were otherwise within normal limits except for a mild elevation of CA724.

**Supplementary Table 1: Blood Routine**

| Name | Result | Unit | Reference range |
| --- | --- | --- | --- |
| WBC | 3.59 | ×10^9/L | 3.50-9.50 |
| LY% | 21.7 | % | 20.0-40.0 |
| MONO% | 9.7 ↑ | % | 3.0-8.0 |
| NEUT% | 67.2 | % | 50.0-75.0 |
| EOS% | 1.1 | % | 0.5-5.0 |
| BASO% | 0.3 | % | 0.0-1.0 |
| LY# | 0.78 ↓ | ×10^9/L | 0.80-4.00 |
| MONO# | 0.35 | ×10^9/L | 0.12-0.80 |
| NEUT# | 2.41 | ×10^9/L | 2.00-7.50 |
| EOS# | 0.04 | ×10^9/L | 0.02-0.50 |
| BASO# | 0.01 | ×10^9/L | 0.00-0.10 |
| RBC | 4.02 | ×10^12/L | 3.50-5.00 |
| HGB | 120 | g/L | 110-150 |
| HCT | 36.7 | % | 35.0-50.0 |
| MCV | 91.3 | fl | 82.0-97.0 |
| MCHC | 327 | g/L | 320-360 |
| MCH | 29.9 | pg | 27.0-32.0 |
| RDW-S | 50.7 ↑ | fl | 39.0-46.0 |
| RDW-C | 15.1 ↑ | % | 0.0-15.0 |
| PLT | 213 | ×10^9/L | 100-350 |
| PCT | 0.2 | % | 0.11-0.28 |
| PDW | 9.8 | fl | 9.0-17.0 |
| MPV | 9.6 | fl | 7.0-13.0 |
| P-LCR | 21.8 | % | 13.0-43.0 |

**Supplementary Table 2: Comprehensive Metabolic Panel**

| Name | Result | Unit | Reference range |
| --- | --- | --- | --- |
| ALT | 14 | U/L | 7-40 |
| TP | 69 | g/L | 60-85 |
| Alb | 41 | g/L | 35-52 |
| A/G | 1.5 |  | 1.0-2.5 |
| PA | 243 | mg/L | 200-400 |
| TBil | 12.1 | μmol/L | 5.1-22.2 |
| DBil | 3.3 | μmol/L | ≤6.8 |
| GGT | 42 | U/L | 7-45 |
| ALP | 100 | U/L | 50-135 |
| AST | 20 | U/L | 13-35 |
| TBA | 1.2 | μmol/L | <10.0 |
| LD | 178 | U/L | 0-250 |
| ChE | 8.4 | kU/L | 5.0-12.0 |
| K | 4.3 | mmol/L | 3.5-5.5 |
| Na | 141 | mmol/L | 135-145 |
| Cl | 107 | mmol/L | 96-111 |
| TCO2 | 28.9 | mmol/L | 20.0-34.0 |
| Ca | 2.3 | mmol/L | 2.11-2.52 |
| cCa | 2.28 | mmol/L |  |
| P | 1.32 | mmol/L | 0.85-1.51 |
| Mg | 0.89 | mmol/L | 0.70-1.10 |
| Cr(E) | 48 | μmol/L | 45-84 |
| Urea | 6.5 | mmol/L | 2.8-7.2 |
| Glu | 5.2 | mmol/L | 3.9-6.1 |
| UA | 273 | μmol/L | 150-357 |
| TC | 5.7 ↑ | mmol/L | <5.2 |
| TG | 1.01 | mmol/L | <1.7 |
| HDL-C | 1.48 | mmol/L | <1.0 |
| LDL-C | 3.66 | mmol/L | <3.4 |
| nonHDL-C | 4.22 | mmol/L | <4.2 |
| ApoA1 | 1.44 | g/L | 1.05-2.05 |
| ApoB | 1.1 | g/L | 0.55-1.30 |
| Lp(a) | 121 | mg/L | 0-300 |
| CRP/hsCRP | 0.67 | mg/L | <3.00 |
| FFA | 370 | μmol/L | 129-769 |
| AMY | 43 | U/L | 36-143 |
| LIP | 50 | U/L | 2-53 |
| CK | 83 | U/L | 24-170 |
| CKMB-mass | 1 | μg/L | ≤5.0 |
| hscTnI | <2.5 | ng/L | ≤34 |
| NT-proBNP | <35 | pg/ml | 0-125 |

**Supplementary Table 3: Coagulation**

| Name | Result | Unit | Reference range |
| --- | --- | --- | --- |
| PT | 11.5 | s | 10.4-12.6 |
| PT% | 109.4 | % | 74.0-120.0 |
| INR | 0.96 |  | 0.86-1.14 |
| Fbg | 3.3 | g/L | 1.80-3.50 |
| APTT | 29.5 | s | 23.3-32.5 |
| APTT-R | 1.09 |  | 0.85-1.20 |
| TT | 17.6 | s | 14.0-21.0 |
| D-Dimer | 0.25 | mg/L FEU | 0-0.55 |

**Supplementary Table 4: Tumor Markers**

| Name | Result | Unit | Reference range |
| --- | --- | --- | --- |
| CA242 | <2.0 | U/ml | ≤25.0 |
| AFP | 2.5 | ng/ml | ≤20.0 |
| CEA | 2 | ng/ml | ≤5.00 |
| CA19-9 | <2 | U/ml | ≤34.0 |
| CA125 | 6.7 | U/ml | ≤35.0 |
| CA72-4 | 10.9 ↑ | U/ml | ≤9.8 |

**Supplementary Table 5: Serum IgG subclasses**

| Name | Result | Unit | Reference range |
| --- | --- | --- | --- |
| IgG1 | 6521 | mg/L | 3941-10444 |
| IgG2 | 5079 | mg/L | 1661-8064 |
| IgG3 | 291 | mg/L | 101-895 |
| IgG4 | 327 | mg/L | 36-2090 |

**Supplementary Table 6: Infectious Disease Screening Panel**

| Name | Result | Unit | Reference range |
| --- | --- | --- | --- |
| HBeAg | Negative(-) 0.41 | S/CO | Negative(-) <1.00 |
| HBeAb | Negative(-) 1.88 | S/CO | Negative(-) >1.00 |
| HBcAb | Negative(-) 0.12 | S/CO | Negative(-) <1.00 |
| HCV-Ab | Negative(-) 0.08 | S/CO | Negative(-) <1.00 |
| TP-Ab | Negative(-) 0.08 | S/CO | Negative(-) <1.00 |
| HIV Ag/Ab | Negative(-) 0.10 | S/CO | Negative(-) <1.00 |

Supplementary figure 1 contains all the pathology results of this patient. Figure 1a to 1c are respectively for the pancreatoduodenectomy specimen, the EUS-FNA specimen in 2025.02, and the fine needle biopsy specimen in 2023.12.


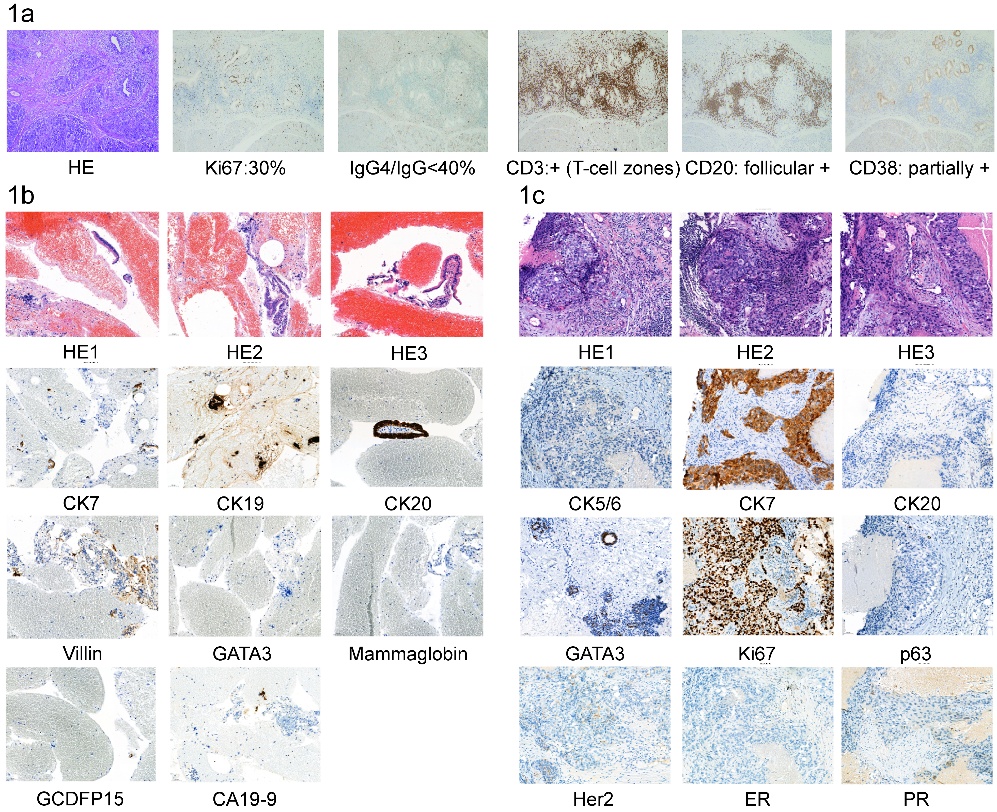

Supplement: Supplementary file 1 [file DataSheet1.docx]
